# Robust Image Stitching for Railway Wagon Inspection in Uncontrolled Outdoor Environments

**DOI:** 10.3390/s26185917

**Published:** 2026-09-19

**Authors:** Alejandro Diaz-Diaz, Francisco Parrilla, Luis M. Bergasa

**Affiliations:** 1 Transportation Department, Indra Company, San Fernando de Henares, 28830 Madrid, Spain; fparrilla@indra.es; 2 Departamento de Electrónica, Universidad de Alcalá (UAH), Alcalá de Henares, 28801 Madrid, Spain; luism.bergasa@uah.es

**Keywords:** European railway checkpoint, image stitching, railway inspection, keypoint filtering, dense feature matching, evaluation framework, industrial computer vision, panoramic imaging, benchmark dataset

## Abstract

Railway wagon inspection is a key enabler of digital railway operations and intelligent asset management that requires a complete view of each wagon side, which a fixed trackside area-scan camera cannot capture in a single frame as the train passes. We present a complete image stitching pipeline that reconstructs each wagon from its sequence of overlapping frames under uncontrolled outdoor conditions: day and night illumination, varied wagon surfaces, background clutter and camera vibration. The pipeline applies seven sequential keypoint filters to dense correspondences produced by a learned dense matcher, followed by robust median displacement estimation and a sequence-level outlier recovery mechanism. We also propose a three-tier evaluation framework that scores a stitching system from its reported geometry alone, without access to its internals, supported by a benchmark dataset of 30 railway wagons across six trains with 262 manually annotated frame pairs. End-to-end evaluation on the benchmark dataset shows that the pipeline recovers accurate displacements where the raw correspondences yield none; no single filter dominates on every measure, with the decisive filter depending on the surface; and the complete system outperforms both a classical sparse-feature baseline and a generic dense-matching baseline. The evaluation framework is publicly released, and the annotated dataset is available on request, to facilitate reproducibility.

## 1. Introduction

The European Railway Checkpoint (ERC) concept, promoted within Europe’s Rail Joint Undertaking (ERJU), supports the digitalisation of rail freight operations [1] and intelligent railway asset management [2] through automated trackside data acquisition infrastructures. Within this framework, image stitching enables the reconstruction of complete, high-resolution views of rolling stock, cargo and freight units from overlapping images captured by wayside systems during train passage. These panoramic representations enhance automated inspection, traceability, cargo identification and anomaly detection, providing reliable data for freight management, operational decision-making and condition-based maintenance.

Such panoramas are also the substrate for downstream automated analysis, such as recognising the standardised wagon (UIC) and container (BIC/ISO 6346) identity codes [3], a task that requires the code surface to appear complete and undistorted in a single image.

Unlike general-purpose image stitching, where arbitrary viewpoint changes require full homography estimation, the fixed-camera railway scenario is constrained: inter-frame motion is a near-pure horizontal translation whose magnitude depends only on wagon speed relative to the camera. This constraint enables a principled 1D displacement estimation approach, but introduces domain-specific failure modes that are absent from standard benchmarks: (i) *mechanical constraints*, such as camera misalignment or vibration, which introduce systematic biases that degrade matching quality; (ii) *repetitive surface texture* (metallic panels, uniform paint), which causes dense matchers to produce large numbers of geometrically inconsistent correspondences; (iii) *degenerate pairs*, where the matcher fails to recover a reliable displacement for a pair (despite continuous wagon motion and full frame overlap), leaving a gap in the displacement sequence.

Existing work on vehicle inspection stitching [4] has demonstrated the value of learned dense matchers for this domain, but relies on semantic background segmentation requiring domain-specific retraining, provides no ablation of individual pipeline components, and evaluates results purely through visual inspection without quantitative metrics. More broadly, standard image stitching and matching benchmarks such as UDIS-D [5], HPatches [6], and the Image Matching Benchmark [7] do not model the domain-specific perturbations of industrial fixed-camera acquisition in real scenarios, making them unsuitable as primary evaluation resources.

This paper makes the following contributions:

1. **Stitching pipeline.** A complete pipeline for panorama composition from a fixed trackside camera (Section 3). It integrates established filtering techniques with a dense matcher into an industrial inspection system that exploits the physical constraints of trackside railway acquisition. Its core is a seven-step hierarchical cascade (geometric ROI, parallax rejection, directional consistency, displacement consistency, vibration suppression, spatial clustering with DBSCAN [8] and geometric verification with MAGSAC++ [9]) that distils the dense correspondences of TinyRoMa [10] into a coherent inlier set, improving the displacement accuracy obtainable from the raw matches of the benchmark (Section 5.2.2). Around it, roll-levelling preprocessing, a robust median displacement estimate, a recovery mechanism that reconstructs pairs the matcher cannot resolve on low-texture surfaces, and alpha blending complete the panorama.
2. **Quantitative evaluation framework.** An objective protocol for scoring fixed-camera sequential stitching (Section 4). It judges a system from the frame offsets it reports using the three-tier metrics defined in this work: Degenerate Rate, Displacement Accuracy against the annotated correspondences, and Sequence Stability, aggregated into a single composite score. To our knowledge it is the first structured quantitative methodology for this setting; each tier is validated by controlled error injection against the failure mode it targets (Section 5.4.1).
3. **Annotated benchmark dataset.** A benchmark of 30 wagons from six trains spanning five surface categories, comprising 403 frames in 373 consecutive pairs, of which 262 are annotated by hand with ten point correspondences each, with  characterised annotation uncertainty (Section 4.1). The evaluation scripts are released publicly and the dataset is available on request so that other systems can be compared under a common protocol on data that reproduces the perturbations of industrial acquisition.

The remainder of this paper is organised as follows. Section 2 reviews related work on image stitching, dense feature matching, and vehicle inspection systems. Section 3 describes the proposed stitching pipeline. Section 4 introduces the evaluation framework and benchmark dataset. Section 5 reports experimental results. Section 6 discusses limitations and future directions. Section 7 concludes the paper.

## 2. Related Work

### 2.1. Image Stitching

The foundational pipeline for automatic image stitching was established by Brown and Lowe [11], who combined invariant feature detection (SIFT [12]), pairwise homography estimation, and multi-band blending into a system capable of assembling unordered photo collections into seamless panoramas. Szeliski [13] provided a comprehensive treatment of the geometric models underlying panoramic composition, from planar projective transformations to cylindrical and spherical warps. These methods assume arbitrary camera viewpoints and estimate full 3×3 homographies, which is appropriate for unconstrained photography but unnecessarily complex for settings where inter-frame motion is geometrically constrained. Spatially varying warps such as as-projective-as-possible stitching with moving DLT [14] relax the single-homography assumption to suppress parallax ghosting, but likewise target unconstrained scenes.

More recently, deep learning has been applied to both alignment and blending stages. Nie et al. [15] proposed UDIS, an unsupervised deep image stitching framework that learns non-rigid warps from image pairs, later extended to UDIS-D [5] with improved handling of parallax. Soltanpour and Joslin [16] and Wang and Yang [17] provide surveys covering both feature-based and learning-based approaches. While these methods advance the state of the art on wide-baseline scenarios, their training and evaluation assume diverse scenes and arbitrary viewpoint changes, unlike the constrained industrial acquisition studied here.

### 2.2. Feature Matching Methods

Classical feature matching relies on hand-crafted detectors and descriptors. SIFT [12] remains a widely used baseline owing to its scale and rotation invariance, though it produces relatively sparse correspondences on textureless or repetitive surfaces. ORB [18] offers a faster alternative with binary descriptors but lower matching accuracy in the presence of viewpoint change.

The emergence of learned features has substantially improved matching quality in challenging conditions. SuperPoint [19] introduced a self-supervised framework that jointly learns an interest point detector and a descriptor, producing semi-dense feature sets suitable for downstream matching. SuperGlue [20] then framed matching itself as a learnable problem, using an attentional graph neural network to jointly establish correspondences and reject unmatchable points; its efficient successor LightGlue [21] retains that accuracy with an adaptive early-exit mechanism.

A separate line of work targets detector-free dense matching. LoFTR [22] introduced a coarse-to-fine transformer architecture that directly produces dense correspondences without explicit keypoint detection, proving especially effective in low-texture regions. DKM [23] cast dense matching as kernelised regression for geometry estimation, and RoMa [10] extended this paradigm with a robust regression-based warp estimator, achieving state-of-the-art results on standard matching benchmarks. Its lightweight variant, TinyRoMa, built on the XFeat [24] feature backbone, retains most of the matching quality at significantly lower computational cost, making it suitable for deployment on industrial edge hardware.

In our domain (metallic wagon surfaces with repetitive paint, rivets, and uniform panels), dense matchers produce thousands of correspondences per frame pair, but a substantial proportion are geometrically inconsistent due to repetitive texture aliasing and 3D parallax from protruding structural elements. This requires a dedicated filtering pipeline that is tailored to the known motion model, rather than generic outlier rejection.

### 2.3. Vehicle and Infrastructure Inspection

Visual inspection of railway infrastructure has been studied primarily for rail surface defect detection. Li and Ren [25] and Deutschl et al. [26] proposed early vision-based systems for detecting rail cracks, corrugation, and surface wear at operational speed. Liu et al. [27] provide a survey of machine-vision methods for railway defect detection. Zhang et al. [28] proposed an object detection model for automatic fault detection on freight train images. Feng et al. [29] developed an automated fastener classification system with cameras capturing sequential images beneath passing coaches. Beyond defect detection, automated reading of wagon and container identity codes from trackside imagery has been addressed with modern object detectors [3]; such recognition operates on the wagon surface itself and benefits directly from a complete, distortion-free panorama of the kind our system produces.

Notably, these systems focus on component-level defect detection or localised recognition rather than full-surface panoramic reconstruction. The task of composing consecutive frames of a moving vehicle into a single seamless image has received comparatively little attention. The most directly related work is that of ElSaer et al. [4], who propose a stitching framework for automobile inspection that combines RoMa with YOLO+SAM semantic background removal and a six-method ensemble outlier filter (Z-score, MAD, sigma clipping, LOF, DBSCAN, HDBSCAN). Their approach produces visually convincing panoramas on three passenger car datasets (8–16 images each), but relies on a segmentation model trained on automotive classes (requiring retraining for new vehicle types) and evaluates results exclusively through qualitative visual inspection, without any quantitative metric. No ablation study is provided to isolate the contribution of individual filtering components.

Our work differs in three respects. First, in the stitching system itself: in place of the trained semantic segmentation and the generic statistical-ensemble outlier filtering of [4], we use a geometric ROI and a hierarchical filter cascade built on the physical priors of the fixed-camera railway setting (a single motion direction, near-constant inter-frame displacement, and a predictable surface band), complemented by a degenerate pair recovery mechanism that completes sequences where matching fails, none of which requires domain-specific training data. Second, we introduce a quantitative three-tier evaluation framework grounded in manually annotated ground truth. Third, we provide a detailed ablation study quantifying the effect of each filtering step on displacement accuracy.

### 2.4. Evaluation of Stitching Quality

The Structural Similarity Index (SSIM) [30] and Normalised Cross-Correlation (NCC) are the most widely used metrics for assessing blended image quality, capturing perceptual consistency in the overlap region [13]. Standard image matching benchmarks such as HPatches [6], the Image Matching Benchmark [7], and the stitching-specific UDIS-D [5] evaluate on diverse, wide-baseline scenes rather than the constrained sequential acquisition studied here.

In the sequential fixed-camera scenario, seam quality metrics alone are insufficient: a system that systematically produces incorrect displacements may still achieve moderate SSIM through alpha blending, while placing structural features at visually incorrect positions, a failure mode we demonstrate empirically in Section 5.4.1. We therefore ground our framework in a displacement accuracy tier anchored to manually annotated ground truth correspondences, rather than in seam appearance, and complement it with sequence-level stability metrics that capture inter-pair consistency. This multi-tier approach enables comparative evaluation across systems and configurations on a benchmark dataset of 30 railway wagon sequences, substantially larger than the three-vehicle datasets used in prior work [4].

## 3. Proposed Stitching System

**Figure 1:**
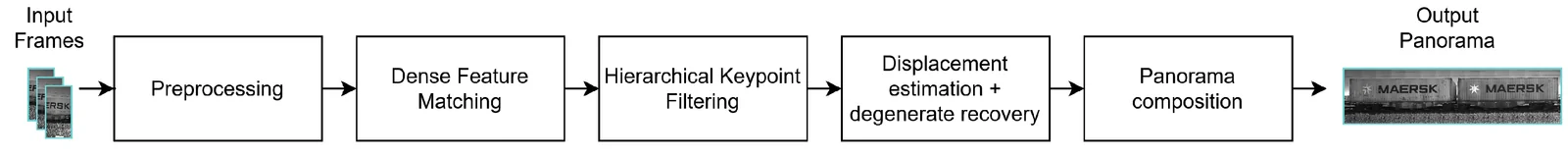
Overview of the proposed stitching pipeline. The hierarchical keypoint filtering stage is detailed in Table 1.

**Figure 2:**
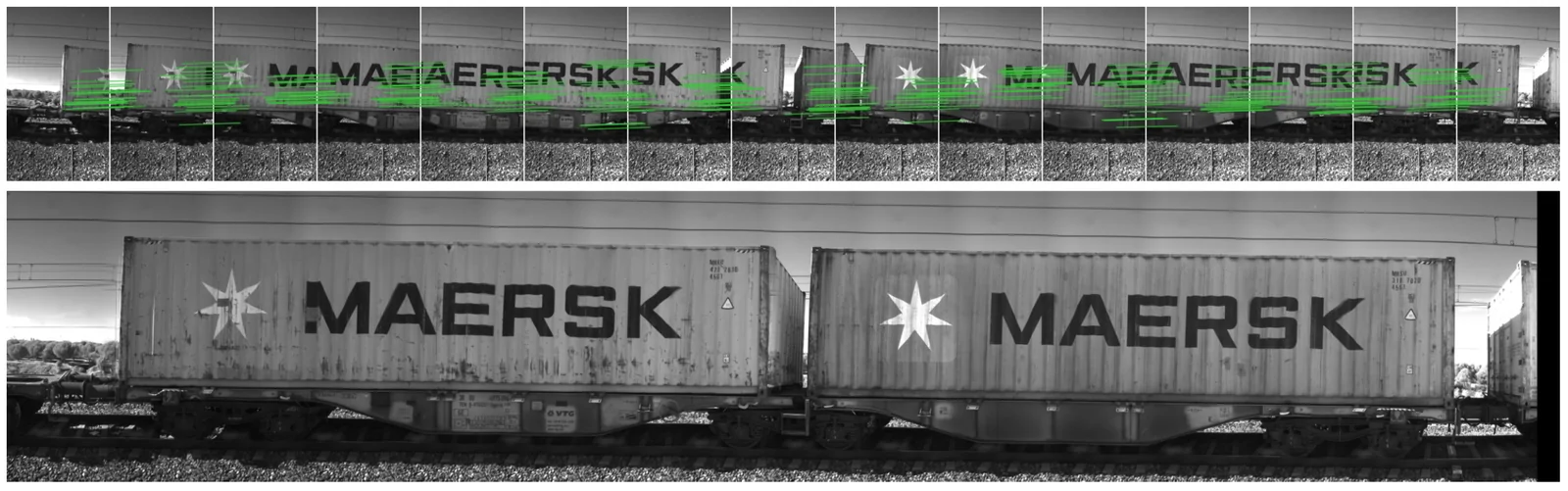
End-to-end result on a representative container wagon. (**Top**): the 15 consecutive input frames, with the surviving post-cascade (filter 7) inlier matches (green) overlaid at each consecutive-pair junction. (**Bottom**): the panorama the system composes from those frames. The per-frame camera roll visible in the inputs is corrected by the levelling step, so the stitched wagon comes out level.

The proposed system takes as input a time-ordered sequence of frames captured by a fixed industrial camera as a freight train passes in front of it, and produces a single panoramic image of each wagon side. The pipeline consists of five main stages (Figure 1): (i) preprocessing, including camera roll correction and border cropping; (ii) dense feature matching between each pair of consecutive frames; (iii) hierarchical filtering of the raw correspondences to retain only geometrically consistent inliers in the overlap zone; (iv) robust displacement estimation and sequence-level outlier recovery; and (v) panorama composition via alpha blending. The following subsections describe each stage in detail. Figure 2 shows the system end to end on a representative wagon.

**Table 1:** Summary of the seven keypoint filters. Each filter receives the output of the previous step and removes a specific category of outlier correspondences.

Step	Filter	Targeted Outlier Type
1	ROI	Background and non-overlap regions
2	Parallax	Near-zero displacement and excessive vertical angle
3	Direction	Motion opposing dominant direction
4	DX Consistency	Large deviation from median Δx
5	Vibration	Excessive vertical jitter
6	Cluster	Spatially isolated correspondences
7	Geometry	Epipolar inconsistency

### 3.1. Problem Formulation and Acquisition Setup

**Figure 3:**
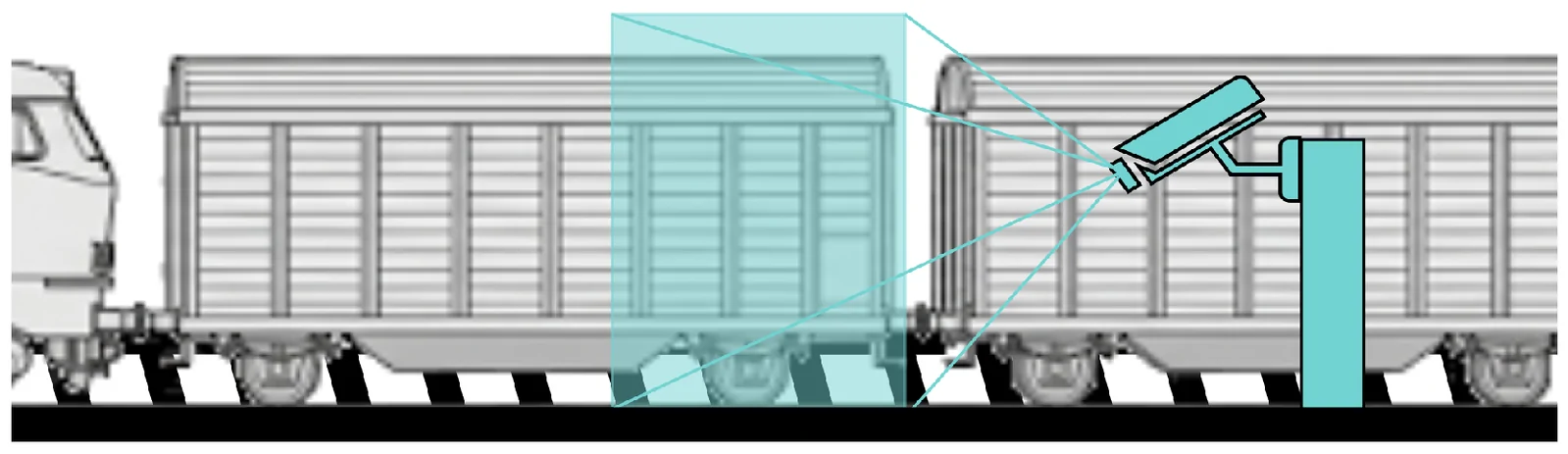
Acquisition setup. A fixed camera mounted beside the track images the passing train, capturing one frame of its field of view (highlighted) at a time.

Let $\left\{F_{0},F_{1},\ldots ,F_{N-1}\right\}$ denote the sequence of *N* frames captured by a single fixed camera as a railway wagon traverses its field of view. The camera is mounted at a fixed position alongside the track, approximately perpendicular to the direction of travel (Figure 3). Due to the continuous motion of the wagon, consecutive frames share a horizontal overlap region whose width depends on the ratio between frame rate and wagon speed.

The objective is to estimate, for each consecutive pair $\left(F_{i},F_{i+1}\right)$, a horizontal displacement $\Delta x_{i}$ such that placing $F_{i+1}$ at offset $\Delta x_{i}$ relative to $F_{i}$ produces a seamless transition in the overlap region. The cumulative canvas position of frame *i* is then $X_{i}=\sum_{j=0}^{i-1}\Delta x_{j}$, with $X_{0}=0$.

Under this formulation, the stitching problem reduces to robust estimation of N−1 scalar displacements from noisy, high-dimensional correspondence sets. The primary challenge is not geometric complexity (the motion model is a simple 1D translation) but rather the reliability of the displacement estimates in the presence of repetitive surface texture, 3D parallax from non-planar wagon elements, and occasional degenerate pairs where the matcher fails to produce a sufficient number of consistent correspondences.

### 3.2. Preprocessing

Prior to feature extraction, each frame undergoes camera roll correction with border cropping; lens distortion correction is supported but not applied in the current deployment.

#### 3.2.1. Camera Roll Correction

The camera mount introduces a small roll that biases the apparent horizontal displacement across the frame, degrading filtering and seam alignment. The pipeline supports two correction modes: an automatic per-frame estimate from the dominant horizon line (Hough transform), or a fixed calibrated angle for installations with stable geometry. All experiments use the fixed angle calibrated for our installation (θ=1.6°), except the end-to-end comparison of Section 5.4, where levelling is itself one of the ablated factors and the reference configuration is reported without it.

#### 3.2.2. Lens Distortion Correction

Radial and tangential lens distortion is not corrected in the current deployment because on-site checkerboard calibration was not feasible. The pipeline supports inserting an undistortion step, which we leave as future work.

### 3.3. Dense Feature Matching

Given a preprocessed frame pair $\left(F_{i},F_{i+1}\right)$, a learned dense feature matcher produces a set of putative correspondences $M_{i}={\left\{\left(p_{k},q_{k}\right)\right\}}_{k=1}^{M}$, where $p_{k}\in F_{i}$ and $q_{k}\in F_{i+1}$ are pixel coordinates.

We use TinyRoMa [10], the lightweight variant of the RoMa dense matcher, as the production configuration. TinyRoMa produces several thousand correspondences per frame pair and offers a favourable trade-off between matching quality and inference time on GPU hardware. On our target surfaces, described in Section 4.1, the raw correspondence set contains a substantial proportion of outliers. These arise from three main sources: (i) repetitive surface texture, which causes the matcher to produce aliased correspondences at incorrect horizontal offsets; (ii) background elements (sky, trackside infrastructure) visible at the frame borders; and (iii) 3D parallax from protruding structural elements (handles, coupling rods, raised lettering) that do not conform to the planar surface assumption.

The following filtering pipeline is designed to progressively eliminate these outlier categories while preserving the geometrically consistent correspondences that lie on the wagon surface within the overlap zone.

### 3.4. Hierarchical Keypoint Filtering

**Figure 4:**
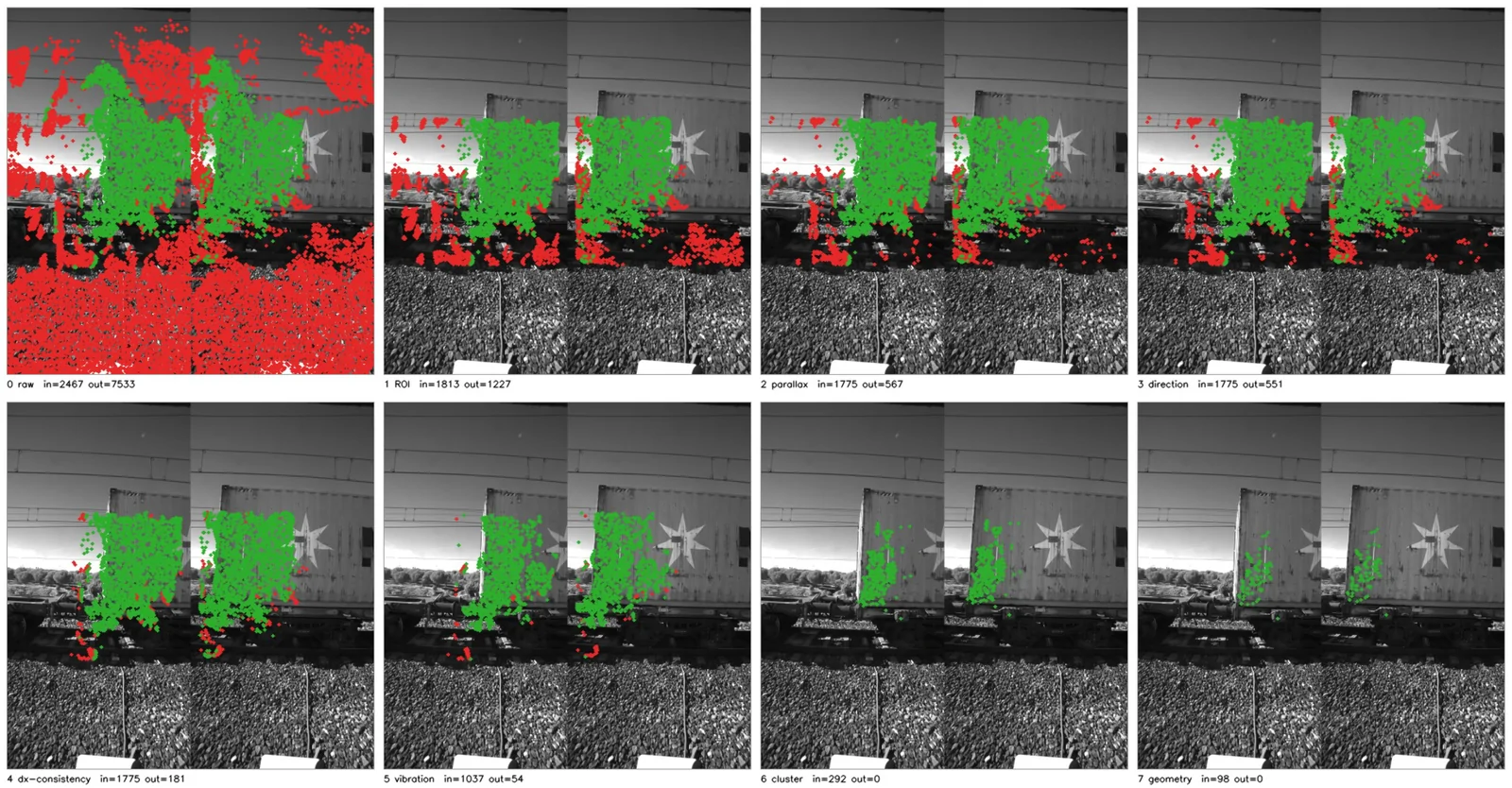
Spatial view of the keypoint-filter cascade on a representative container pair (frame $F_{i}$ (left), $F_{i+1}$ (right)), one panel per cumulative step (0 raw to 7 geometry). Each surviving match is coloured by the displacement label defined above: **green** if its horizontal displacement agrees with the ground truth ($|\Delta x-\Delta x^{\mathrm{gt}}|\;\leq 15\%\;|\Delta x^{\mathrm{gt}}|$), **red** otherwise.

Seven filters are applied sequentially to the raw correspondence set (Figure 4). Each filter operates on the output of the previous one, progressively refining the inlier set $M_{i}^{\left(s\right)}$ at each step *s*. The final set $M_{i}^{\left(7\right)}$ is used for displacement estimation. Table 1 summarises the cascade and the outlier category each step targets. Several of these filters exploit physical constraints that are specific to the fixed-camera railway inspection scenario: the single, consistent motion direction of the wagon within a pass, the approximately constant inter-frame displacement within a sequence, and the predictable vertical position of the wagon surface in the frame. These domain-specific priors enable aggressive outlier rejection that would not be applicable in a general-purpose stitching pipeline.

#### 3.4.1. ROI Filter

The ROI filter (Figure 4, panel 1, “ROI”) exploits a structural regularity of the setting: wagons have roughly consistent dimensions (vehicle height is bounded by the railway loading gauge) and pass at a fixed distance, so the wagon surface always falls within a predictable vertical band of the frame. It restricts correspondences to a fixed rectangular region in relative coordinates: vertical boundaries (fractions of the frame height) exclude the ground, sky and permanent foreground structures (rail supports, cable trays), with a small horizontal margin trimming the frame edges. Unlike the trained semantic segmentation it replaces (Section 2), it requires no training data, at the cost of not adapting to atypical geometries, not a limiting factor in our deployment. It removes the largest share of outlier correspondences in the cascade (Section 5.2).

#### 3.4.2. Parallax Filter

The parallax filter (Figure 4, panel 2, “parallax”) operates in two stages. First, correspondences with near-zero horizontal displacement ($|\Delta x_{k}|<\tau_{\min}$) are removed, as these typically originate from static background elements or surfaces at effectively infinite depth. Second, correspondences whose motion vector forms an angle exceeding $\alpha_{\max}$ with the horizontal axis are rejected. Under the 1D translation model, valid correspondences should exhibit Δy≈0; a significant vertical component indicates a match on a surface at a different depth than the wagon plane, where 3D parallax produces apparent vertical motion.

#### 3.4.3. Direction Filter

The dominant motion direction (left or right) is the sign of the median horizontal displacement (Figure 4, panel 3, “direction”), and correspondences whose displacement opposes it are rejected. The direction is estimated from the post-parallax matches rather than the raw set, as they are more reliable on low-texture surfaces, where the raw matches may contain many aliased correspondences.

#### 3.4.4. DX Consistency Filter

This filter (Figure 4, panel 4, “dx-consistency”) removes correspondences whose horizontal displacement $\Delta x_{k}$ deviates excessively from the current consensus. The threshold is computed dynamically as $\tau =\max(r\cdot |\tilde{\Delta x}|,\;\tau_{\min})$, where *r* is a relative factor, $\tilde{\Delta x}$ is the median displacement of the current set, and $\tau_{\min}$ is a minimum absolute threshold that prevents the criterion from becoming degenerate when $|\tilde{\Delta x}|$ is small. The filter targets correspondences on elements at depths different from the main wagon surface, which produce systematic displacement offsets due to perspective.

#### 3.4.5. Vibration Filter

Mechanical vibration (Figure 4, panel 5, “vibration”) of the camera or the passing wagon can introduce small vertical oscillations in the correspondence positions. The vibration filter removes correspondences whose vertical displacement $\Delta y_{k}$ deviates by more than $\tau_{j}$ pixels from the median $\tilde{\Delta y}$ of the current set. This step stabilises the vertical consistency of the inlier set, reducing noise in the downstream displacement estimate.

#### 3.4.6. Cluster Filter

The cluster filter (Figure 4, panel 6, “cluster”) applies the DBSCAN algorithm [8] to the displacement vectors $\left(\Delta x_{k},\Delta y_{k}\right)$ of the surviving correspondences, in pixel units, with a neighbourhood radius of 2 px and a minimum cluster size of 20 matches. The largest cluster is retained; correspondences belonging to smaller clusters or classified as noise are rejected. This step captures spatially coherent groups of matches that share a consistent displacement, effectively removing isolated outliers that passed the previous filters by chance. As a safeguard, if DBSCAN classifies all points as noise (which occurs when the displacement distribution is too dispersed for reliable clustering, as can happen on empty wagon surfaces with matches at varying depths), the filter is skipped entirely, deferring outlier removal to the geometry filter. Neither value is critical: the radius matches the displacement agreement expected on a rigid surface, the minimum size sits well below the correspondences that reach this step, and sweeping the radius from 0.5 to 32 px and the minimum size from 5 to 160 keeps mean T2 over the annotated pairs within 0.79–0.81.

#### 3.4.7. Geometry Filter

The final filter (Figure 4, panel 7, “geometry”) enforces global geometric consistency: genuine surface correspondences are all induced by the same rigid camera–wagon motion, whereas the outliers that survive the earlier per-match heuristics (typically matches on protruding 3D elements, or chance mismatches with a plausible horizontal displacement) violate that single geometry. We estimate a fundamental matrix over the surviving matches with MAGSAC++ [9], which replaces the hard inlier/outlier decision of standard RANSAC by marginalising the hypothesis score over a range of noise scales up to a maximum threshold, here 0.5 px at a confidence of 0.999; varying that threshold from 0.25 to 4 px moves mean T2 by less than 0.01. This suits the domain because the boundary between a surface inlier and a slight-depth outlier is gradual rather than sharp. If MAGSAC++ does not converge (e.g., too few surviving matches for a stable fit), the system falls back to standard RANSAC [31] so the pair still yields an estimate rather than being dropped. This is the strictest purity check in the cascade: as the ablation shows (Section 5.2), it leaves a small, geometrically coherent inlier set rather than raising average accuracy.

### 3.5. Displacement Estimation

The per-pair horizontal displacement $\Delta x_{i}$ is the median (not the mean, for robustness to surviving outliers) of the horizontal components of the final inlier set $M_{i}^{\left(7\right)}$. If too few inliers survive, the pair is flagged as degenerate and handled by the recovery mechanism (Section 3.6).

The displacement is applied as a rigid per-pair translation, yielding a vertical seam: the small real dependence of the apparent displacement on the image row (the acquisition geometry residual of Section 4.1.3) cannot be estimated reliably from the per-pair match scatter. Fitting a per-row polynomial over the surviving matches confirms this: across the benchmark it lowers the residual RMS only from 26.6 px at degree zero to 16.9 px at degree three, less than the 23.5 px median annotation uncertainty of the dataset (Section 4.1.3), while the per-pair fits vary by two orders of magnitude and carry no consistent relation to the measured gradient. The gain is therefore within our own measurement noise, and we keep the rigid model.

### 3.6. Degenerate Pair Recovery

A pair is *degenerate* when the pipeline retains too few inliers, or returns a near-zero displacement while the rest of the sequence is clearly moving: a matching failure on low-texture or highly repetitive surfaces, not an actual halt, since freight trains move at near-constant speed through the acquisition window. A second category, *statistical outlier pairs*, produces a non-empty inlier set but an inconsistent displacement (e.g., a direction-reversed estimate on a symmetric surface), detected when a pair’s overlap deviates markedly from the sequence median. Both are corrected by replacing the estimate with the average of the nearest valid neighbours, which the slow, smooth speed variation makes a reliable surrogate, completing the sequence, and hence the panorama, even when individual pairs fail. Left uncorrected, degenerate pairs would collapse consecutive frames; we quantify the module’s effect in Section 5.3.

### 3.7. Panorama Composition

The panorama is assembled incrementally on a dynamically expanding canvas, each frame $F_{i}$ placed by rigid translation at its cumulative position $X_{i}$ (Figure 2). Overlaps are merged by linear alpha compositing: a validity mask marks real content, and a linear ramp transitions the blend weight across a blend width adapted to each pair’s actual overlap, so the transition never extends beyond the jointly valid region. Pairs still degenerate after recovery are placed at the sequence median displacement, a plausible if not pixel-accurate continuation. The system outputs the panorama together with a frame_offsets.json of cumulative positions, the sole input required by the evaluation framework (Section 4).

## 4. Evaluation Framework

We propose a quantitative three-tier evaluation framework designed for the fixed-camera sequential stitching scenario. The framework treats the stitching system as a *black box*: the only required output is a file recording the cumulative horizontal canvas position of each frame. No access to internal algorithm state is needed. T1 and T3 operate exclusively on this offset file; T2 additionally reads the ground truth annotations supplied with the benchmark itself. No frame images are required, which enables any system (regardless of its internal architecture) to be evaluated on the same benchmark.

### 4.1. Benchmark Dataset

#### 4.1.1. Data Collection

**Figure 5:**
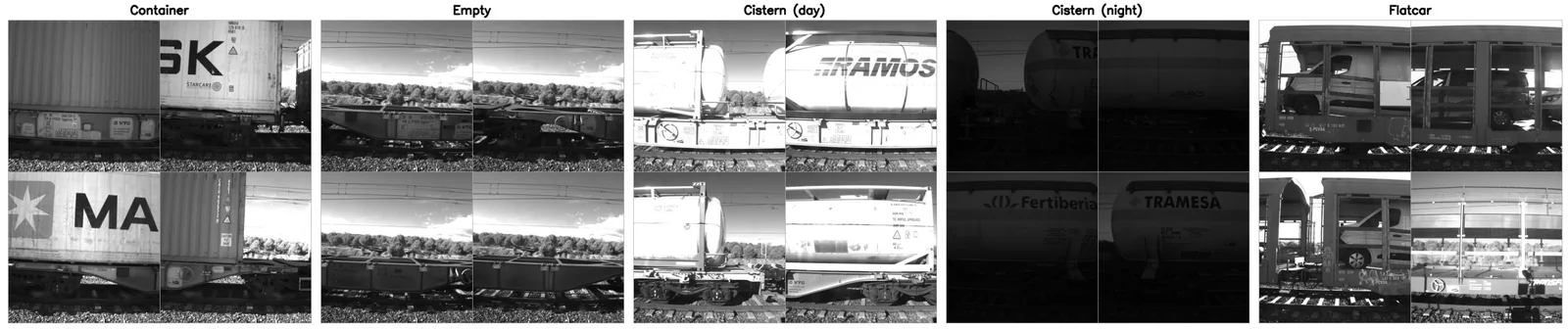
Four representative frames per surface category (centre-cropped to square tiles), spanning the five categories evaluated in Section 5.2 (Table 2): closed containers, empty wagons, day and night cisterns, and flatcars carrying road vehicles.

The benchmark dataset consists of 30 railway wagon sequences drawn from six trains acquired under operational conditions at fixed trackside camera installations, comprising 403 frames that form 373 consecutive pairs. Acquisition conditions span daytime, nighttime, and mixed illumination scenarios. Wagon types include closed containers, open flatcars, and cisterns, providing surface texture diversity (Figure 5).

**Table 2:** Raw match composition per surface category (representative frames in Figure 5): per-pair means of displacement inliers and outliers under the labelling rule of Section 5.2.1.

Category	Raw Matches	Inliers	Outliers	Inlier %
Container	10,000	2189	7811	21.9%
Empty	10,000	604	9396	6.0%
Cistern (day)	10,000	1203	8797	12.0%
Cistern (night)	10,000	1249	8751	12.5%
Flatcar	10,000	1531	8469	15.3%
All (mean)	10,000	1342	8658	13.4%

#### 4.1.2. Ground Truth Annotation

**Figure 6:**
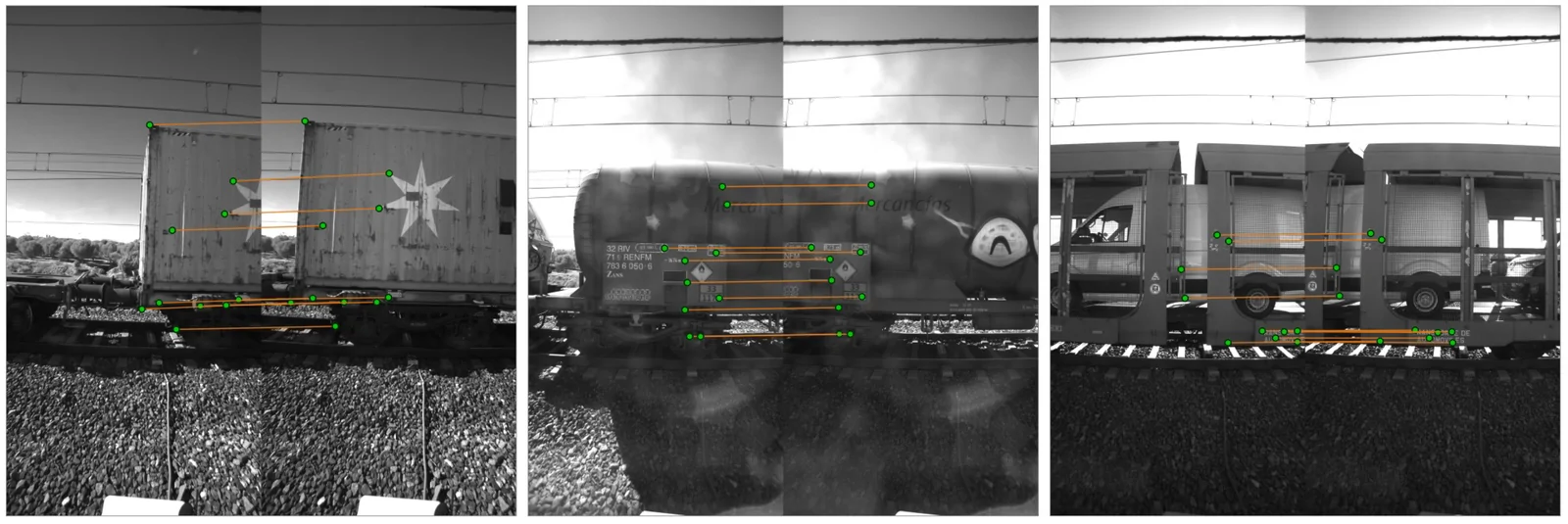
Ground truth annotation for three representative pairs (container, cistern, vehicle-carrying flatcar). The ten manually annotated correspondences (green) are drawn as lines linking frame $F_{i}$ (left) to $F_{i+1}$ (right). Points are placed on flat, planar wagon surfaces and avoid edges, depth discontinuities and background; the pair ground truth $\Delta x^{\mathrm{gt}}$ is their median horizontal shift (Equation (1)).

For each pair of consecutive frames, 10 point correspondences were manually annotated in the overlap zone (Figure 6). All annotations were made on the frames as acquired, before any geometric preprocessing, so the ground truth carries the same lens distortion and camera roll as the released images. Annotators selected points on flat, planar wagon surfaces (panel faces, smooth metal areas) and avoided edges, corners, cables, coupling elements and any surface at a structural depth different from the main wagon plane: because the system estimates a single 1D horizontal displacement per pair, points on non-coplanar surfaces would mix depth planes and yield a non-representative ground truth. The ground truth displacement of pair *i* is the median of its 10 per-point horizontal displacements,

(1) $$\Delta x_{i}^{\mathrm{gt}}={\mathrm{median}}_{k}\left(q_{k,x}-p_{k,x}\right),$$

where $\left(p_{k,x},q_{k,x}\right)$ are the *x*-coordinates of the *k*-th correspondence in frames $F_{i}$ and $F_{i+1}$.

The spread of those 10 displacements is itself an indirect measure of the non-translational component of the apparent inter-frame transformation (camera roll residual, perspective, parallax); we take its standard deviation $\sigma_{i}$ as the pair’s *annotation quality* and re-annotated any pair with $\sigma_{i}>50$ px. Across the 262 annotated pairs the median σ is 23.5 px, with only two pairs (0.8%) marginally above 50 px (51.3 and 53.7 px) on night-time surfaces where no better-distributed point set could be identified; train_02 is the weakest subset ($\sigma_{\mathrm{median}}=40.7$ px) owing to very dark, low-feature images, and is retained with that caveat. The first 10 pairs of each wagon are annotated (wagons with fewer pairs in full), for 262 pairs in total, 70% of the 373 in the benchmark; surface type and illumination are constant throughout a pass, so further pairs would repeat the same condition.

#### 4.1.3. Annotation Validity and Limitations

Manual pixel-level measurements on a reference wagon revealed a linear vertical gradient in seam ghosting of approximately +45 px per 1000 px downward. This is not a frame-to-frame rotation (the wagon translates rigidly along the track) but a geometric residual the 1D model cannot represent: the wagon side is not perfectly fronto-parallel to the sensor (a small mount tilt, in the same family as the measured camera roll), producing a perspective foreshortening that grows with vertical position, compounded by residual lens distortion and 3D relief on protruding elements. A single horizontal displacement is therefore exact at one image row and accumulates error above and below it.

This residual stems from acquisition geometry rather than the estimator, so it is the same for every system using the 1D translation model. It therefore does not affect T2’s validity (Section 4.3) as a **comparative** metric, because T2 isolates the one component that varies between systems: estimator accuracy. A score of T2 = 1.0 means the estimated displacement is optimal within the translation model, not that visual ghosting is zero.

### 4.2. Tier 1: Degenerate Rate (T1)

T1 detects pairs where the stitching system effectively collapses two consecutive frames onto each other, creating an invisible seam at the cost of duplicating the surface content. T1 is expected to be 1.0 under normal motion and degrades only when the system fails on a moving sequence. In practice, T1 is a nearly binary signal.

(2) $$T1=1-\frac{n_{\mathrm{degen}}}{n_{\mathrm{total}}},$$

where $n_{\mathrm{degen}}$ is the number of pairs for which $|\Delta x_{i}|\;\leq 10$ px while the sequence median $|\overset{\;˜}{x\Delta }|>50$ px, and $n_{\mathrm{total}}$ is the total number of pairs in the sequence.

### 4.3. Tier 2: Displacement Accuracy (T2)

T2 quantifies the error between the system’s estimated displacement $\Delta x_{i}^{\mathrm{sys}}$ and the annotated ground truth $\Delta x_{i}^{\mathrm{gt}}$:

(3) $$e_{i}=\left||\Delta x_{i}^{\mathrm{sys}}|-|\Delta x_{i}^{\mathrm{gt}}|\right|,$$

(4) $$s_{i}=\left\{\begin{array}{cc} 1.0 & \mathrm{if}\;e_{i}/\left|\Delta x_{i}^{\mathrm{gt}}\right|\leq \alpha_{\mathrm{lo}},\\ 1-\frac{e_{i}/\left|\Delta x_{i}^{\mathrm{gt}}\right|-\alpha_{\mathrm{lo}}}{\alpha_{\mathrm{hi}}-\alpha_{\mathrm{lo}}}\; & \mathrm{if}\;\alpha_{\mathrm{lo}}<e_{i}/\left|\Delta x_{i}^{\mathrm{gt}}\right|\leq \alpha_{\mathrm{hi}},\\ 0.0 & \mathrm{if}\;e_{i}/\left|\Delta x_{i}^{\mathrm{gt}}\right|>\alpha_{\mathrm{hi}}, \end{array}\right.$$

where $\alpha_{\mathrm{lo}}=0.05$ and $\alpha_{\mathrm{hi}}=0.20$ define the tolerance band as fractions of $|\Delta x_{i}^{\mathrm{gt}}|$. The pair score $s_{i}=1.0$ within the inner tolerance (errors ≤5% of ground truth) accounts for annotation uncertainty. The wagon-level T2 score is the mean over all annotated pairs. Because the annotations are made on the frames as acquired and under the same 1D translation model (Section 4.1.2), T2 measures agreement with the best displacement that model admits rather than absolute panorama alignment; the acquisition geometry residual of Section 4.1.3 is common to every 1D system and does not enter the comparison.

### 4.4. Tier 3: Sequence Stability (T3)

T3 measures the temporal consistency of displacement estimates across the frame sequence:

(5) $$T3=\max\;\left(0,\;1-\frac{\mathrm{CoV}(\Delta x^{\mathrm{sys}})}{{\mathrm{CoV}}_{\max}}\right),\;\mathrm{CoV}\left(\Delta x\right)=\frac{\sigma (\Delta x)}{\left|\mu (\Delta x)\right|},$$

where CoV is the coefficient of variation computed over the moving pairs of the sequence (|Δx|>50 px, excluding stationary and degenerate pairs) and ${\mathrm{CoV}}_{\max}=0.50$ is a normalisation constant calibrated to operational data. T3 penalises sequences where the estimated displacement fluctuates excessively relative to its mean, which would produce visible width irregularities in the panorama. It captures a failure dimension that the wagon-mean T2 dilutes: a single mis-estimated pair leaves T2 nearly unchanged yet collapses T3 through the inter-pair variance (Section 5.4.1).

### 4.5. Composite Score

The composite score aggregates the three tiers via a weighted geometric mean:

(6) $$C={T1}^{w_{1}}\cdot {T2}^{w_{2}}\cdot {T3}^{w_{3}},$$

with weights $\left(w_{1},w_{2},w_{3}\right)=\left(0.1,0.6,0.3\right)$, which sum to one so that the exponents need no renormalisation. The weights are priority indicators rather than empirically calibrated coefficients: T2 carries the highest weight as the primary accuracy metric and the tier with the widest discriminative range (Section 5.4.1), T3 (sequence stability) is a secondary consistency signal, and T1 receives minimal weight because it is nearly always 1.0 for sequences with valid motion. A formal sensitivity analysis of the weighting is done in Section 5.4.1. The geometric mean has the property that a score of zero on any single tier collapses the composite to zero, ensuring that catastrophic failures are never masked by strong performance on other dimensions.

## 5. Experiments

### 5.1. Experimental Setup

Unless stated otherwise, all experiments use TinyRoMa (romatch v0.1.2) as the feature matcher, run on a single NVIDIA Tesla T4 (NVIDIA Corporation, Santa Clara, CA, USA) with CUDA 12.1. The full filter pipeline (steps 1–7, Section 3.4) is applied in its production configuration. The ablation of Section 5.2 therefore runs at the production levelling angle (θ=1.6°, Section 3.2) while the annotations are on the frames as acquired (Section 4.1.2); Section 5.4 measures what that difference costs end to end, the same correction shifting the composite by +0.001 in the mean and leaving the median unchanged.

### 5.2. Ablation Study of the Filtering Pipeline

We conduct a formal ablation study to quantify the individual contribution of each filter step in the cascade, over the full annotated benchmark (30 wagons, 262 frame pairs, Section 4.1) grouped into the five surface categories of Table 2. Ground truth annotations (Section 4.1.2) label every match as a displacement inlier or outlier and are used to evaluate T2 at every step (a match with individual horizontal displacement estimate *d* is an inlier if $|d-\Delta x_{\mathrm{gt}}|\leq 0.15\;|\Delta x_{\mathrm{gt}}|$, and an outlier otherwise). To account for the stochasticity of the dense matcher (GPU non-determinism) and of the MAGSAC++ geometry filter (randomised sampling), each pair is processed in 10 independent runs; all reported T2 values are means across these runs, with per-cell standard deviation ≤0.015.

#### 5.2.1. Filter Precision Analysis

Every inlier or outlier label is defined on the *horizontal* displacement alone and does not assess vertical alignment, a scope that matters when interpreting steps 5–7 below. This lets us count, after every step, how many matches survive and how many of them are inliers (Figure 4). Treating outlier removal as the positive task, a removed outlier is a true positive (TP), a removed inlier a false positive (FP), and a surviving outlier a false negative (FN).

The dense matcher returns a fixed budget of 10,000 correspondences per pair (a TinyRoMa configuration parameter), and only a minority of them are displacement inliers. That minority varies by almost a factor of four across surfaces (Table 2). These starting proportions, not the filters alone, govern what each step can achieve: removing one outlier out of a hundred is a different problem from removing one out of two.

Table 3 follows the surviving set through the cascade. The first four filters perform the bulk of the purification: they crush the outlier population from 8658 to 189 matches per pair while preserving the inliers almost intact (1342 → 1022, 76% retained), raising the inlier fraction of the survivors from 13% to 84%. The last three filters behave oppositely: they shrink the set from 1211 to 71 matches, but now the removed matches are overwhelmingly inliers (FP ≫ TP at each step), because only 189 outliers remain to be found when they begin.

Normalising removals by the total match count would conflate a filter’s discriminative ability with the prevailing class balance, so we report *class-conditional* rates, computed from counts summed over all 262 pairs (thus weighting each pair by its match count): outlier **Recall**=TP/(TP + FN), the share of entering outliers removed; **InlierLoss**=FP/(FP + TN), the share of entering inliers wrongly removed; and **Precision**=TP/(TP + FP), the share of removed matches that were genuine outliers. Because Precision is itself bounded by the class balance, we list beside it the **Prior**=Outliers/Matches, the fraction of entering matches that are outliers (Table 3). Reading Precision *against* the Prior, rather than on an absolute scale, is what makes it interpretable (Table 4).

The ROI and Parallax filters account for 6503 and 1416 of the outliers removed, more than the remaining five steps combined. This follows from cascade position rather than from stronger discrimination: they act on populations that are 86.6% and 66.6% outliers, whereas later steps face at most 41.0%. This is why the filters are better compared by how far each operates above its prior than by the volume it removes.

Steps 1–4 operate well above their prior: each removes outliers at a Precision far higher than the outlier base rate it faces (Direction, 96.0% versus a 41.0% prior; DX Consistency, 91.4% versus 32.6%), discarding between eleven and one hundred outliers per inlier sacrificed (FP/TP ≤0.09). Inlier loss is negligible for Parallax, Direction and DX Consistency (≤2.9%); the ROI crop is the only one of the four to shed an appreciable share of *labelled* inliers (19.5%). Steps 1–4 succeed because they discriminate directly on the horizontal-displacement axis the median estimator relies on. The one exception, ROI’s high inlier loss, stems from two causes: catenary and background matches alias with the wagon’s motion and get mislabelled as inliers, and the fixed ROI band also discards genuine inliers near the top of containers.

Steps 5–7 tell a different story: their Precision collapses to *essentially the prior*, 20.4% versus a 15.6% prior (Vibration), 11.1% versus 12.3% (Cluster), 14.5% versus 13.8% (Geometry). Steps 5–7 select on criteria orthogonal to horizontal displacement (vertical consistency, spatial clustering, global geometric agreement), an axis the per-pair median does not use; this is why they do not improve T2 beyond the step-4 plateau (Section 5.2.2). Their value lies instead in robustness: on clean pairs the median is already correct, but on hard, low-inlier pairs the residual outliers steps 1–4 leave behind can still corrupt it, and removing them is precisely what these steps do (confirmed end-to-end in Section 5.4).

#### 5.2.2. Displacement Accuracy Progression

Section 5.2.1 characterised *what* each filter removes; we now measure how that translates into displacement accuracy. Recall (Section 4.3) that T2 scores each pair by the relative error of its estimated displacement against ground truth, saturating to 1.0 below 5% error and to 0.0 above 20%, with the wagon score the mean over its pairs. Table 5 reports mean T2 after each cumulative filter step.

All five categories start at T2 = 0.000, and no single filter dominates this progression: the score improves through the early filters up to its best value at step 5 (Vibration), whose gain over the previous step is already negligible, unlike the earlier ones. Steps 6 and 7 not only fail to improve T2 but, in every case except the container wagon, produce a slight decrease.

This raises the question of whether the pipeline could simply be truncated at step 4. The metric T2 cannot settle it: residual outliers do not move it on clean pairs, but on low-inlier-ratio pairs they may be numerous enough to corrupt it. We resolve this in Section 5.4, comparing both variants on the full benchmark under the three-tier framework.

### 5.3. Evaluation of Degenerate Pair Recovery

The matcher and the seven-filter cascade are run once per pair; the recovery module is then applied to the resulting displacement sequence, so the comparison between recovery disabled and enabled is deterministic and isolates the module’s effect.

The recovery module addresses two distinct failures, as defined in Section 3.6. A pair is *degenerate* when the filtering pipeline cannot recover a usable displacement and the estimate collapses to zero. A *statistical outlier* is a pair that does produce a displacement, but one inconsistent with the rest of the sequence. Both are repaired by the same step (replacing the affected estimate with the average displacement of the nearest reliable neighbours), but they differ in how the repair is validated: a degenerate pair has no prior estimate, so its filled value is checked directly against ground truth (Table 6), whereas a statistical outlier already carries a (wrong) estimate and is therefore assessed separately, as a secondary safeguard (below). Across the benchmark, the pipeline produces 72 degenerate pairs and 28 statistical outliers, for 100 corrected pairs in total over 23 of the 30 wagons.

#### 5.3.1. Recovery Accuracy

We validate the filled values directly against ground truth on the 46 degenerate pairs that carry an annotation. The recovered displacement matches the true value to a median of 20.5 px, or 2.4% of the displacement magnitude, with 36 of the 46 pairs within a 5% relative error. This is below the median annotation uncertainty of the dataset itself (23.5 px, Section 4.1.3) and within the inner 5% tolerance of the evaluation framework. This is due to the low acceleration profiles of trains, which keep the wagon speed nearly constant throughout the pass (median within-wagon CoV of 1.9%). That subset is not a random sample of the failures: its degenerate rate is 17.6%, against 23.4% on the unannotated pairs, so it is the easier half. What the recovered error depends on, however, is the length of the failure run rather than the surface, and on that axis the subset is representative.

#### 5.3.2. Sensitivity to Gap Length

Recovery accuracy depends on how many pairs fail consecutively, since a longer blackout pushes the nearest reliable neighbours further away. Isolated failures and runs of two are recovered to within a 2% median relative error, whereas the longest blackout observed (a run of six consecutive failures on a uniform cistern) degrades to a 7.8% median error. That worst case falls inside the annotated subset; outside it no run exceeds three consecutive failures, so the errors reported above span the full range of gap lengths present in the benchmark.

#### 5.3.3. Statistical Outliers and Failure Modes

The same neighbour-averaging step also re-estimates the 28 statistical outliers. This is beneficial on average: on the 23 that are annotated the median error falls from 35% (the flagged estimate) to 2.4%, and 19 of the 23 improve. But, unlike filling a genuine gap, it is not uniformly safe.

Recovering a degenerate pair from valid neighbours is therefore the robust, primary function of the module; re-estimating statistical outliers is a secondary safeguard that helps in aggregate but cannot exceed the quality of the surrounding sequence. Overall, recovery completes all 23 affected sequences, cutting the median per-wagon displacement standard deviation σ(Δx) from 362.5 to 20.4 px.

### 5.4. End-to-End Evaluation on the Benchmark Dataset

We evaluate the complete pipeline (matcher, seven-filter cascade and degenerate pair recovery) end to end on the 30-wagon benchmark with the three-tier framework, contrasting five configurations of the same system (Table 7). Because end-to-end matching is stochastic, each configuration is run five times and we report the mean over wagons (per-repetition standard deviation ≤0.01); for the composite we additionally report the median over the 30 wagons, which is informative because a single wagon behaves pathologically, as analysed below.

**Table 7:** End-to-end evaluation of five configurations of the system on the 30-wagon benchmark. All configurations are evaluated without roll levelling (θ=0°) except where indicated, so that the comparison isolates the effect of the filtering and recovery stages; levelling is shown separately to be marginal. T2, T3 and the composite are reported as wagon mean/wagon median: the means of the Filters 1–7 configurations are dragged by a single aliasing wagon, whereas the medians are robust to it. T1 is reported as a mean only, since it saturates; with recovery enabled it is exactly 1.0 for 98.9% of the wagon scores (Table 8a), so its median carries no information.

Configuration	T1	T2	T3	Composite
No filters	1.000	0.000/0.000	0.967/1.000	0.000/0.000
Filters 1–4	1.000	0.946/0.972	0.922/0.931	0.943/0.954
Filters 1–7	0.999	0.920/0.990	0.919/0.942	0.923/0.972
Filters 1–7 + levelling 1.6°	1.000	0.925/0.984	0.927/0.952	0.924/0.972
Filters 1–7, no recovery	0.835	0.700/0.689	0.756/0.825	0.705/0.725

**Table 8:** Sensitivity of the composite score. (**a**) Discriminative range of each tier over the three recovery-enabled configurations of Table 7 (89 of their 90 wagon scores; the aliasing wagon, whose T2 collapses, is excluded); *Saturated* is the share scoring 1.0 to three decimals. (**b**) Median composite of three configurations of Table 7 under each sweep; *n* is the grid size (5×5 weight combinations, six tolerance bands, five ${\mathrm{CoV}}_{\max}$ values, five cutoffs). The ranges pool different parameter settings, so they overlap even though Filters 1–7 scores above Filters 1–4, and both above the no-recovery configuration, in every individual combination.

**(a) Discriminative range of each tier**					
**Tier**	**Weight**	**Saturated**	**IQR**	**Range**	
T1 (Degenerate Rate)	0.1	98.9%	0.000	0.980–1.000	
T2 (Displacement Accuracy)	0.6	34.8%	0.071	0.077–1.000	
T3 (Sequence Stability)	0.3	0.0%	0.061	0.359–0.990	
**(b) Composite under the parameter sweeps**					
**Parameter Swept**	**Range**	***n***	**Filters** **1–4**	**Filters** **1–7**	**No** **Recovery**
Composite weights	$w_{2}$ 0.4–0.8	25	0.953–0.974	0.963–0.989	0.699–0.752
T2 tolerance band	(0.02,0.10)–(0.10,0.40)	6	0.858–0.976	0.910–0.982	0.670–0.767
T3 constant ${\mathrm{CoV}}_{\max}$	0.25–1.00	5	0.936–0.968	0.949–0.984	0.653–0.750
T3 moving-pair cutoff	20–200 px	5	0.954	0.972	0.700–0.723

Four results follow. First, **the keypoint-filtering cascade is what makes the system usable at all**: without it the displacement accuracy collapses to zero (T2 =0.000, composite =0.000), because the raw dense correspondences do not yield a reliable per-pair displacement. The first four filters already recover the bulk of the performance (composite 0.954 median).

**Figure 7:**
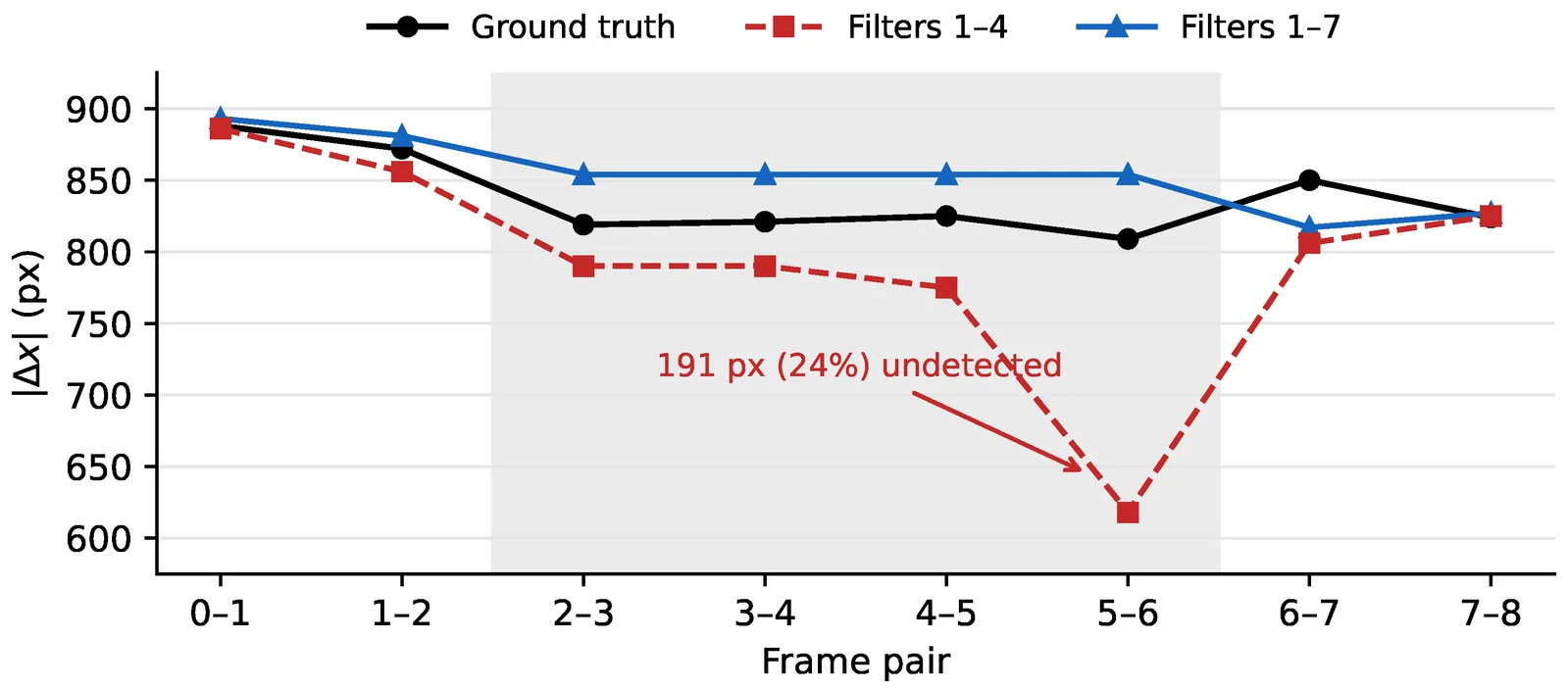
Displacement sequence of a day cistern (train_04, wagon_05) under the truncated and the full cascade, against ground truth; medians over the five repetitions, with shading marking the pairs the recovery module repaired under the full cascade. The full cascade is not more accurate pair by pair: on pairs 2–3 and 3–4 its repaired value sits further from ground truth than the truncated estimate. What it avoids is the undetected error at pair 5–6.

Second, **the refinement filters 5–7 add outlier robustness, not average accuracy**. The direction here matches Table 5, computed on the same wagons and the same annotated pairs: average T2 falls slightly (0.946→0.920), and the improvement appears only in the median. The absolute levels differ because the two tables score different systems: Table 5 scores the cascade alone, so a pair it cannot resolve scores zero, whereas here the recovery module has already repaired them. In the wagon median the full cascade improves modestly but consistently over the truncated one on every tier (composite 0.954→0.972), concentrated on the harder wagons where residual outliers would otherwise corrupt the median. The one exception is a flatcar with a repetitive load surface, where the matcher aliases onto a pattern repeat and the per-pair direction becomes unstable across repetitions (Section 3.4.3); this single wagon drags the full cascade’s *mean* below its median. A per-pass majority vote on direction would fix it, which we leave as future work. Figure 7 shows the case on the wagon where the two configurations differ most. Truncating at step 4 leaves pair 5–6 estimated at 618 px against a true 809 px, an error that no stage of the pipeline flags and that therefore reaches the panorama, whereas under the full cascade the same pair is repaired to within 45 px in every repetition.

Third, **camera-roll levelling has only a marginal effect here**: the production 1.6° correction shifts the composite by +0.001 in the mean and leaves the median unchanged, within noise. Levelling corrects the *vertical* motion while T2 measures the horizontal offset, and the cascade already absorbs this installation’s moderate roll; it remains a design provision for deployments with pronounced roll (Section 3.2).

Fourth, **degenerate pair recovery is essential to reliability**: disabling it degrades all three tiers at once (median composite 0.972→0.725), the end-to-end counterpart of the controlled-injection check (Section 5.4.1). It also confirms that T1 and T3 (flat across the recovery-enabled configurations) do discriminate once their target failure modes are present: they are inactive guardrails on an already-corrected signal, not redundant padding. The mechanism itself is analysed in Section 5.3.

#### 5.4.1. Validation of the Evaluation Framework

**Figure 8:**
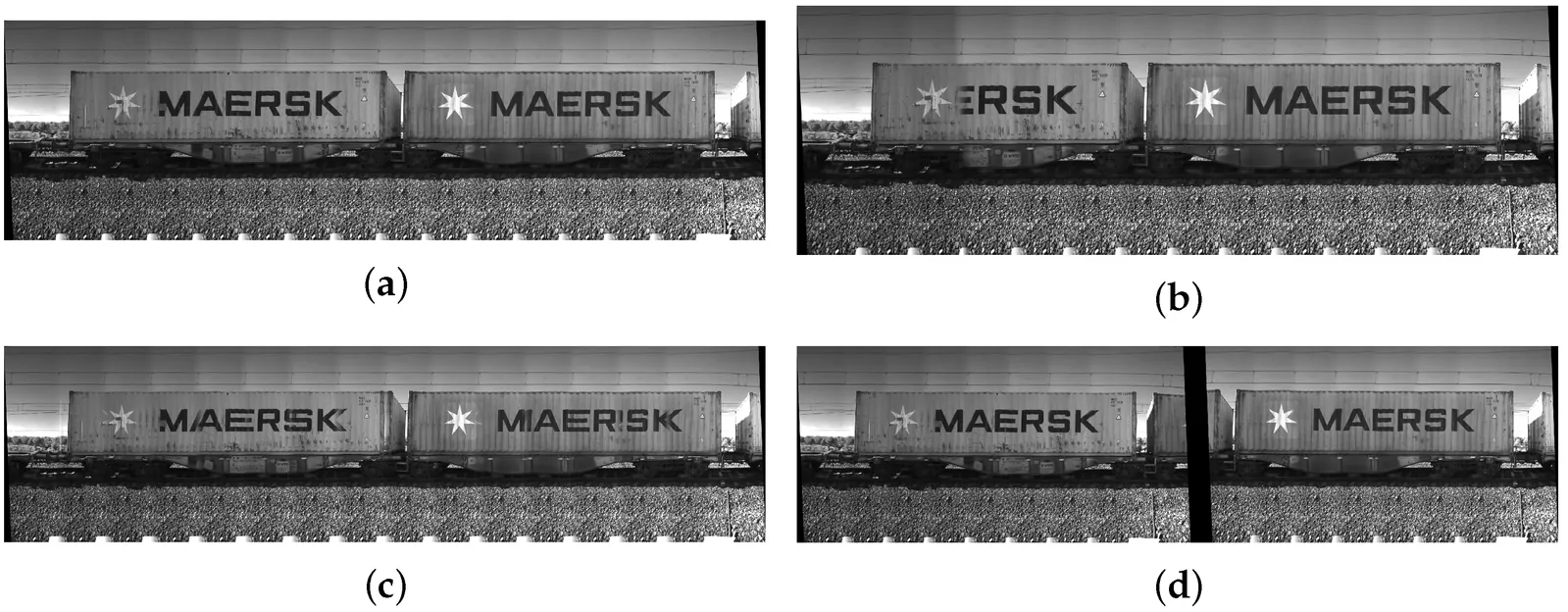
Panoramas produced for each injected error of Table 9, de-rolled and cropped for display: (**a**) reference, correct stitch (composite 0.996); (**b**) collapse of two pairs: surface content is lost (the container legend reads “ERSK”); T1 =0.86; (**c**) systematic bias (+15%): a plausible-looking train, yet the geometry is 15% wrong; T2 =0.15; (**d**) single outlier (×3): a gross tear opens at the affected seam; T3 =0.07.

We validate by controlled error injection that each tier responds to the failure mode it is designed to measure and that the tiers are mutually non-redundant. Starting from a correctly stitched reference wagon, we inject three parametric error families into the reported offsets and re-score: *collapse* (forcing pairs to zero displacement, the degenerate mode), *systematic bias* (scaling every displacement by a constant factor), and a *single outlier* (multiplying one interior pair). Figure 8 and Table 9 report the response. Each error degrades primarily the tier it targets, and the off-diagonal entries establish that the tiers are independent: a systematic bias collapses T2 while leaving T1 and T3 unchanged, a uniform scaling preserves the coefficient of variation exactly, and a single outlier collapses T3, through the inter-pair variance, while T2 (a mean over annotated pairs) is barely affected. No tier subsumes another.

#### Sensitivity Analysis of Tier Weighting

The weights follow how much each tier actually separates configurations (Table 8a): T1 scores 1.0 for almost every wagon, so it orders nothing and serves only as the guardrail of the geometric mean, whereas T2 spans the widest range and contributes 83% of the variation of lnC against 17% for T3, measured as the spread each weighted log term contributes to the sum. We then re-scored the five configurations of Table 7 under four sweeps of the free parameters—the composite weights, the T2 tolerance band, the T3 normalisation constant ${\mathrm{CoV}}_{\max}$ and the cutoff that selects the moving pairs entering it—over the ranges of Table 8b. The ranking is unchanged in all 41 combinations, the only exchange being between the two levelling variants, which the composite separates by at most 0.002 and which swap wherever T3 is given more weight against T2, consistent with levelling trading displacement accuracy for sequence stability. Absolute values shift by up to 0.12, as expected of a comparative score.

### 5.5. Comparison Against Baseline Pipelines

We contrast the complete system with two standard alternatives (Table 10), each scored by the same three-tier framework (five repetitions, per-cell std ≤0.02): a classical sparse matcher (SIFT) and a dense matcher (TinyRoMa), each combined with two motion models, a full homography and the 1D translation the proposed system assumes, each evaluated on the full frame and restricted to the wagon ROI. All share identical inputs; only the matching, motion model and outlier rejection stages vary.

On the full frame every baseline collapses (composite ≤0.11) regardless of motion model: dominated by background and repetitive texture, the largest consistent set locks onto near-zero displacements rather than the wagon’s motion. Restricting to the ROI recovers part of the accuracy (to 0.31–0.45), confirming the region prior is essential, yet the proposed system roughly doubles that again (to 0.92): its physically motivated filters remove the structured outliers that generic geometric verification cannot, and the recovery module completes the sequences they cannot resolve. On the ROI, TinyRoMa outscores SIFT under both motion models, justifying the dense matcher over classical sparse features.

Table 11 reports the cost per frame pair on the Tesla T4 of Section 5.1. Within the cascade, the MAGSAC++ geometry step alone accounts for 42 of the 46 ms, the other six filters and the median estimate staying below 5 ms together. Either way the motion model is a minor term, 7% of the matching cost for the full homography and 0.1% for the 1D estimate: throughput is set by the matching stage, not by the geometry.

## 6. Discussion

### 6.1. Scope and Validity of the 1D Translation Model

The pipeline models inter-frame motion as a pure horizontal translation. This is a domain-specific simplification, not a general stitching solution: it is justified by the constrained geometry of fixed-camera railway acquisition, where it proves both sufficient and robust (Section 5.4).

### 6.2. Distribution of Composite Scores

The per-wagon composite scores are largely bimodal (Table 7): under the full cascade, 28 of the 30 wagons fall in [0.80,1.00] while the failures collapse to very low values (the unfiltered configuration to 0, the aliasing wagon to 0.16), with the middle range sparsely populated. This reflects the underlying estimation problem: the matcher either converges to the correct displacement for a given surface or fails outright, with little middle ground. This confirms the succeed-or-fail character of the system. The pattern is not absolute, however: disabling recovery leaves pairs only partially corrected and pushes several wagons into intermediate scores, and one wagon sits at 0.56. Because the distribution is bimodal, the composite doubles as an automatic quality gate: wagons in the low cluster can be flagged for manual review, the rest processed with confidence.

### 6.3. Limitations and Future Directions

#### 6.3.1. Frame Preprocessing in the Benchmark

The benchmark frames are released as acquired, without geometric preprocessing: they retain lens distortion and a small camera roll. The ground truth annotations were made on these same as-acquired frames, so both effects are absorbed into the ground truth and the resulting residual is a property of the dataset rather than of the estimator. The deployed system corrects the roll at runtime (Section 3.2) but leaves lens distortion uncorrected; the end-to-end reference configuration is evaluated without levelling, so that estimates and annotations share the same frame geometry. A future release with distortion- and roll-corrected frames, annotated on those frames, would remove this bias and could lower the error floor measured by T2.

#### 6.3.2. Dataset Coverage

The benchmark dataset contains 262 annotated pairs from 30 wagons across six trains. While this represents a significant increase over comparable published datasets in the vehicle inspection domain [4], it covers only a subset of the full operational variability in wagon types and lighting, and it contains no precipitation or fog sequences, so the robustness reported here does not extend to those conditions. Expanding the dataset with adverse-weather sequences and additional wagon geometries would increase the generalisability of the reported results.

#### 6.3.3. ROI Adaptability

The geometric ROI is a fixed band, which works well in this deployment but assumes a fixed camera position and a consistent wagon region; it does not adapt when the camera placement or the wagon height varies. In such settings a dynamic, content-aware approach such as semantic segmentation would be the ideal solution. We deliberately avoid it here because of its retraining and runtime cost, which the fixed ROI eliminates, but where those costs are acceptable a segmentation-based ROI would be the more robust choice.

#### 6.3.4. Domain-Specific Matcher

The filtering cascade exists because the raw correspondences from TinyRoMa, used off the shelf, contain a large fraction of outliers on these surfaces. The baseline comparison (Section 5.5) makes this concrete: generic homography RANSAC over the raw matches recovers only part of the accuracy that the cascade attains. Fine-tuning or retraining a dense matcher on railway imagery would attack the problem at its source, reducing outliers before filtering and potentially improving accuracy on the hardest, low-texture surfaces. This requires domain-specific training data and additional compute, so we leave it as future work.

#### 6.3.5. Evaluation Framework Scope

The framework has two known limitations. The first is intrinsic to the tiers: T1 is effectively binary and, within the success regime, the composite is driven mostly by T2, which accounts for 83% of its variation (Table 8). The second is more fundamental. The evaluator scores the *estimated per-pair geometry*; it consumes the reported frame offsets (and, for T2, the ground truth annotations), not the rendered panorama, so downstream compositing, blending and any within-frame warp fall outside its scope, and two systems reporting the same offsets receive the same score regardless of how they render. This is faithful for systems in the 1D-translation model, as ours is, but the ideal evaluation would be genuinely end-to-end, i.e., scoring the final panorama itself, agnostic to how it was produced. That remains an open problem, because there is no ground truth panorama to compare against and appearance-based seam metrics do not reliably capture misalignment under blending (Figure 8c). A metric anchored to ground truth geometry rather than visual coherence is the natural next step, which we leave to future work.

## 7. Conclusions

We have presented a complete image stitching system that reconstructs each wagon side from a fixed industrial camera, supporting automated inspection, traceability, cargo identification and anomaly detection, together with a three-tier quantitative evaluation framework and an annotated benchmark dataset.

The hierarchical keypoint filtering pipeline raises displacement accuracy (T2) from zero on the raw correspondences to between 0.70 and 0.98 across surface types; no single filter dominates on every measure, and the decisive step depends on the surface (Section 5.2). The complete system outperforms both a classical SIFT+RANSAC pipeline and a generic dense-RANSAC baseline (Section 5.5). The degenerate pair recovery module completes the sequences the matcher cannot resolve (19.3% of the benchmark), and disabling it lowers the median composite from 0.97 to 0.73, confirming its role in reliability (Section 5.4).

The three-tier evaluation framework (Degenerate Rate, Displacement Accuracy, Sequence Stability) assesses a system from its reported frame offsets alone, with no access to internal algorithm state, and controlled error injection confirms that each tier responds to the failure mode it targets and that the tiers are non-redundant (Section 5.4.1). Its analysis also establishes T2 as a valid comparative metric between systems within the 1D model, up to a constant acquisition geometry residual it cannot represent.

The end-to-end comparison demonstrates a practical application of the framework: it assigns a composite of exactly zero to the unfiltered configuration (flagging a structural failure rather than a small penalty) and still separates configurations that differ only by a refinement step, consistently across repetitions. It also localises behaviour to individual wagons: on a single flatcar with a repetitive load the per-pair direction estimate fails through aliasing and the metric correctly collapses there while remaining high elsewhere, an attribution that motivates a pass-level direction estimate as future work.

Future work, detailed in Section 6, includes fine-tuning a domain-specific dense matcher to reduce outliers at the source, a content-adaptive ROI for less constrained installations, expanding and geometrically correcting the benchmark dataset, and extending the framework towards a panorama-level end-to-end metric.

## Figures and Tables

**Table 3:** Cascade mechanics: per-pair means over the 262 GT pairs of the after-filter state (Matches, Inliers, and Outliers remaining) and of what each step removes (Rmvd, TP, FP, FN, as defined in Section 5.2.1). Counts are absolute.

Step	Filter	Matches	Inliers	Outliers	Rmvd	TP	FP	FN
0	(raw)	10,000	1342	8658	–	–	–	–
1	ROI	3235	1081	2154	6765	6503	262	2154
2	Parallax	1800	1062	738	1435	1416	19	738
3	Direction	1562	1052	510	238	229	9	510
4	DX Consistency	1211	1022	189	351	321	30	189
5	Vibration	716	628	88	495	101	394	88
6	Cluster	320	276	44	396	44	352	44
7	Geometry	71	63	8	249	36	213	8

**Table 4:** Filter discrimination, aggregated over all 262 GT pairs (counts summed over pairs, then converted to rates; metrics defined in the text). Recall: outliers removed/entering; InlierLoss: inliers removed/entering; Precision: true outliers among removals, to be read against the Prior (outlier fraction entering the step); FP/TP: inliers lost per outlier removed.

Step	Filter	Recall	InlierLoss	Precision	Prior	FP/TP
1	ROI	75.1%	19.5%	96.1%	86.6%	0.04
2	Parallax	65.7%	1.7%	98.7%	66.6%	0.01
3	Direction	31.0%	0.9%	96.0%	41.0%	0.04
4	DX Consistency	62.9%	2.9%	91.4%	32.6%	0.09
5	Vibration	53.4%	38.5%	20.4%	15.6%	3.90
6	Cluster (DBSCAN)	49.8%	56.1%	11.1%	12.3%	8.03
7	Geometry (MAGSAC++)	81.8%	77.2%	14.5%	13.8%	5.88

**Table 5:** Mean T2 (Displacement Accuracy) after each cumulative filter step, per surface category. Values are means over 10 repeated runs; per-cell standard deviation is ≤0.015. Bold marks the per-column maximum.

Step	Active Filters	Container	Empty	Cistern (Day)	Cistern (Night)	Flatcar
0	None	0.000	0.000	0.000	0.000	0.000
1	+ROI	0.583	0.000	0.231	0.320	0.306
2	+Parallax	0.929	0.467	0.627	0.533	0.570
3	+Direction	0.944	0.787	0.684	0.693	0.680
4	+DX Consistency	0.975	0.832	0.711	0.731	0.798
5	+Vibration	0.977	**0.838**	**0.712**	**0.733**	**0.801**
6	+Cluster (DBSCAN)	0.977	0.836	0.707	0.729	0.792
7	+Geometry (MAGSAC++)	**0.977**	0.833	0.705	0.718	0.790

**Table 6:** Degenerate pair prevalence and recovery by surface category. *Degenerate* counts pairs for which the pipeline produced no reliable displacement; *Recovered* is the number of those subsequently filled by the module; *Rec. Error* is the median relative error of the recovered displacement against ground truth, over the degenerate pairs that carry an annotation.

Category	Pairs	Degenerate	Recovered	Rec. Error
Container (day)	69	3 (4.3%)	3/3	1.9%
Empty (day)	98	22 (22.4%)	22/22	2.0%
Cistern (day)	77	18 (23.4%)	18/18	3.1%
Cistern (night)	37	11 (29.7%)	11/11	4.0%
Flatcar (day)	92	18 (19.6%)	18/18	2.1%
Overall	373	72 (19.3%)	72/72	2.4%

**Table 9:** Construct-validity check by controlled error injection on a reference wagon. Bold marks, in each row, the tier that the injected error is designed to affect.

Injected Error	T1	T2	T3	Composite
None (reference)	1.00	1.00	0.99	0.996
Collapse (2 pairs)	**0.86**	0.78	0.99	0.85
Systematic bias (+15%)	1.00	**0.15**	0.99	0.32
Single outlier (×3)	1.00	0.89	**0.07**	0.42

**Table 10:** Baseline comparison on the 30-wagon benchmark. “Full” uses the whole frame, “ROI” the fixed wagon band; composite is reported as wagon mean/median.

Configuration	T1	T2	T3	Composite
SIFT + homography (full)	1.00	0.06	0.96	0.11/0.00
SIFT + translation (full)	1.00	0.05	1.00	0.10/0.00
TinyRoMa + homography (full)	1.00	0.00	1.00	0.00/0.00
TinyRoMa + translation (full)	1.00	0.00	1.00	0.00/0.00
SIFT + homography (ROI)	0.98	0.25	0.92	0.33/0.30
SIFT + translation (ROI)	0.98	0.24	0.94	0.31/0.28
TinyRoMa + homography (ROI)	0.92	0.50	0.74	0.45/0.52
TinyRoMa + translation (ROI)	0.92	0.48	0.76	0.42/0.47
Proposed (complete system)	1.00	0.92	0.92	0.92/0.97

**Table 11:** Mean runtime per frame pair, over the 373 consecutive pairs of the benchmark. The two motion model rows are measured over the same raw correspondence sets and are alternatives to each other, not additional stages.

Stage	Time (ms)	Share
Dense matching (TinyRoMa)	307	79%
Preprocessing (roll and cropping)	34	9%
Filter cascade and displacement	46	12%
Complete pipeline	388	100%
*Motion model, over the same correspondences*		
Full homography (RANSAC)	20.4	
1D translation (consensus)	0.4	

## Data Availability

The evaluation framework is publicly available at https://github.com/AlejandroDiazD/railway-stitching-benchmark (accessed on 10 September 2026), together with usage instructions and citation. The full benchmark dataset is available on reasonable request from the corresponding author, or following the procedure indicated in the repository; it is not publicly redistributable due to commercial restrictions on the source imagery. The stitching system code is proprietary and not publicly available.
